# Effect of Closed Kinetic Chain Exercise With Customized Knee Brace on Pain and Functional Performance in Patients With Bilateral Medial Compartment Knee Osteoarthritis

**DOI:** 10.7759/cureus.89674

**Published:** 2025-08-09

**Authors:** Akshanda Dhumale, Sandeep Shinde

**Affiliations:** 1 Department of Musculoskeletal Sciences, Krishna College of Physiotherapy, Krishna Vishwa Vidyapeeth (Deemed to be University), Karad, IND

**Keywords:** exercise, knee, muscle strength, osteoarthritis, quality of life

## Abstract

Background: Knee osteoarthritis (OA) is a degenerative condition often seen in the elderly, influenced by factors like joint alignment, mechanics, and muscle strength. It affects functional performance in daily activities due to pain and reduced mobility. Closed kinetic chain exercise (CKCE) can enhance joint stability, strength, and proprioception, while a customized knee brace helps offload the medial compartment by correcting malalignment. This study explores the combined effect of CKCE and a customized brace on pain and function in individuals with bilateral medial compartment knee OA.

Objective: This study aimed to evaluate the effectiveness of a structured CKCE program combined with a customized valgus unloader knee brace in reducing pain and improving range of motion (ROM), muscle strength, and overall functional performance in individuals with bilateral medial compartment knee OA, compared to conventional physiotherapy alone.

Methods: A total of 204 participants were initially recruited and equally allocated into Group A and Group B (102 participants each) using the envelope method. However, eight participants were lost to follow-up, resulting in 196 participants (98 in each group) who completed the study. Over six weeks, the patients engaged in conventional exercises and CKCE with a customized knee brace. The Visual Analogue Scale (VAS), ROM, manual muscle testing (MMT), and Knee Injury and Osteoarthritis Outcome Score (KOOS) were utilized for evaluating the results.

Results: Both groups showed significant improvements post-intervention, but Group B demonstrated superior outcomes across all measures. Pain scores (VAS) at rest and during activity decreased more in Group B (p<0.0001). ROM and MMT significantly improved, particularly in knee flexion and extension (p<0.0001). KOOS domains, namely, pain, symptoms, activities of daily living (ADL), sports/recreation, and quality of life, also showed greater improvements in Group B (p<0.0001), reflecting enhanced functional performance.

Conclusion: The combination of CKCE and a customized knee brace was more effective than conventional physiotherapy alone in reducing pain and improving functional outcomes in patients with bilateral medial compartment knee OA. This integrated intervention enhances joint mechanics, promotes muscular strength, and supports a better quality of life. CKCE with bracing should be considered a viable conservative treatment strategy in the management of medial knee OA.

## Introduction

Knee osteoarthritis (OA), commonly referred to as degenerative joint disease, is primarily caused by the gradual deterioration and loss of articular cartilage due to prolonged wear and tear [1]. OA is the second most prevalent rheumatologic condition and the leading joint disease in India, with a reported prevalence ranging from 22% to 39%. It is more common in females than in males, with incidence increasing significantly with age. Radiological evidence of the condition is observed in approximately 70% of women over the age of 65 [2]. In neutrally aligned limbs, the medial compartment of the knee bears 60-70% of the load during weight-bearing activities, making it more frequently affected than the lateral compartment [3]. Varus deformities further increase the load on the medial compartment, accelerating cartilage degeneration and worsening the condition [4,5]. Pain in knee OA can be triggered by altered biomechanics, including malalignment, body size, and muscular strength, all of which influence the magnitude and direction of knee joint loading. Abnormal mechanical loading is believed to contribute to tissue degradation and the onset of pain [6]. Gait and balance are also compromised in knee OA. Proper function of the sensorimotor system, including proprioceptive accuracy and muscle control, is essential for maintaining balance and producing a smooth, stable gait. Quadriceps dysfunction, commonly seen in knee OA, may impair balance and mobility, thereby reducing overall functional performance [7]. Individuals with knee OA often exhibit reduced strength in the muscles surrounding the joint, which may or may not be associated with muscle atrophy, pain, or swelling. This muscular impairment can significantly affect quality of life and contribute to limitations in functional activities [8]. Functional performance of the knee fundamentally involves its ability to support body weight, allow for bending and movement, and enable activities such as walking, running, and jumping [9].

Over time, numerous therapeutic interventions have demonstrated efficacy in managing knee OA. One such intervention is closed kinetic chain exercise (CKCE). However, its specific impact on medial compartment knee OA remains underexplored. CKCE involves movements in which the distal segments of the body are fixed and unable to move freely against external resistance. This coordinated, segmental motion increases the demand for muscular co-contraction, thereby stabilizing and controlling movement across the joints of the kinetic chain [10,11]. In addition to enhancing muscle strength, CKCE may also improve joint position sense. It is well-established that CKCE improves both muscle strength and proprioceptive function by activating a greater number of muscle spindles and joint proprioceptors, thereby better preparing patients for activities of daily living (ADL) [12]. Because CKCE is performed in a weight-bearing position, it enhances knee stability, particularly in the medial compartment, by strengthening the quadriceps, hamstrings, and hip abductors. Improved muscle strength helps maintain joint integrity, reduces pressure on the cartilage, and redistributes forces more evenly, potentially unloading the affected medial side [13,14]. Bilateral medial compartment OA presents unique biomechanical and functional challenges. However, there is currently no standardized CKCE protocol specifically tailored for this condition [15].

In conjunction with exercise, braces serve as an effective non-pharmacological intervention to improve knee alignment. The primary objective of using a knee brace is to alter load distribution across the affected joint compartment. By correcting joint instability and malalignment, braces help reduce mechanical stress on the symptomatic area [16]. Valgus unloader braces alleviate pain by decreasing the load on the medial compartment of the knee. They apply an external valgus force to counteract the typical varus alignment observed in medial compartment OA. This corrective force is transmitted through adjustable straps or condylar pads, while opposing forces from the brace's upper and lower supports act above and below the joint. Together, these forces help redistribute pressure more evenly across the knee joint, thereby decreasing mechanical strain on the medial side [17]. However, there is limited evidence specifically evaluating the effects of CKCE in individuals with bilateral medial compartment knee OA. The human body integrates sensory input from vision, somatosensory feedback, and the vestibular system to maintain balance. This integration results in coordinated muscle contractions in response to the gathered information. CKCE plays a key role in enhancing postural control by promoting synchronized muscular activity and joint stability [18,19].

The present study introduces a novel, comprehensive rehabilitation strategy that combines CKCE with the use of a customized knee brace to optimize outcomes in individuals with bilateral medial compartment knee OA. This integrated protocol addresses the entire lower kinetic chain, that is, the hip, knee, and ankle, while the brace provides external support to correct malalignment, enhance joint stability, and offload the symptomatic medial compartment. By simultaneously targeting muscular and mechanical factors, the intervention aims to reduce joint stress and promote more balanced load distribution during movement. Structured into four progressive stages, namely, activation, stabilization, strengthening, and functional performance, the protocol ensures a systematic and individualized progression through each phase of recovery [20]. This study aimed to evaluate the effect of CKCE with a customized knee brace on pain reduction and functional performance improvement in individuals with bilateral medial compartment knee OA.

## Materials and methods

This comparative study was conducted at Krishna College of Physiotherapy, Krishna Vishwa Vidyapeeth (Deemed to be University), Karad, India, after obtaining ethical clearance from the Institutional Ethics Committee of Krishna Vishwa Vidyapeeth (Deemed to be University) (approval number: 028/2023-2024). Eligible participants, determined based on the inclusion and exclusion criteria, were enrolled after providing written informed consent and receiving an explanation of the study's purpose and procedures. Both male and female individuals aged between 45 and 65 years were recruited. All participants had a clinical diagnosis of bilateral medial compartment knee OA. Additional inclusion criteria required participants to be within the specified age range and have a body mass index (BMI) between 25 and 35 kg/m², classifying them as overweight or obese. Radiographic confirmation of knee OA as Grade 1 or Grade 2 according to the Kellgren-Lawrence grading scale was also mandatory. Participants were excluded if they had a history of fractures, recent surgical procedures, severe cardiovascular or neurological conditions, or visual impairments. Individuals who were not willing to participate were also excluded.

Treatment protocol

**Figure 1:**
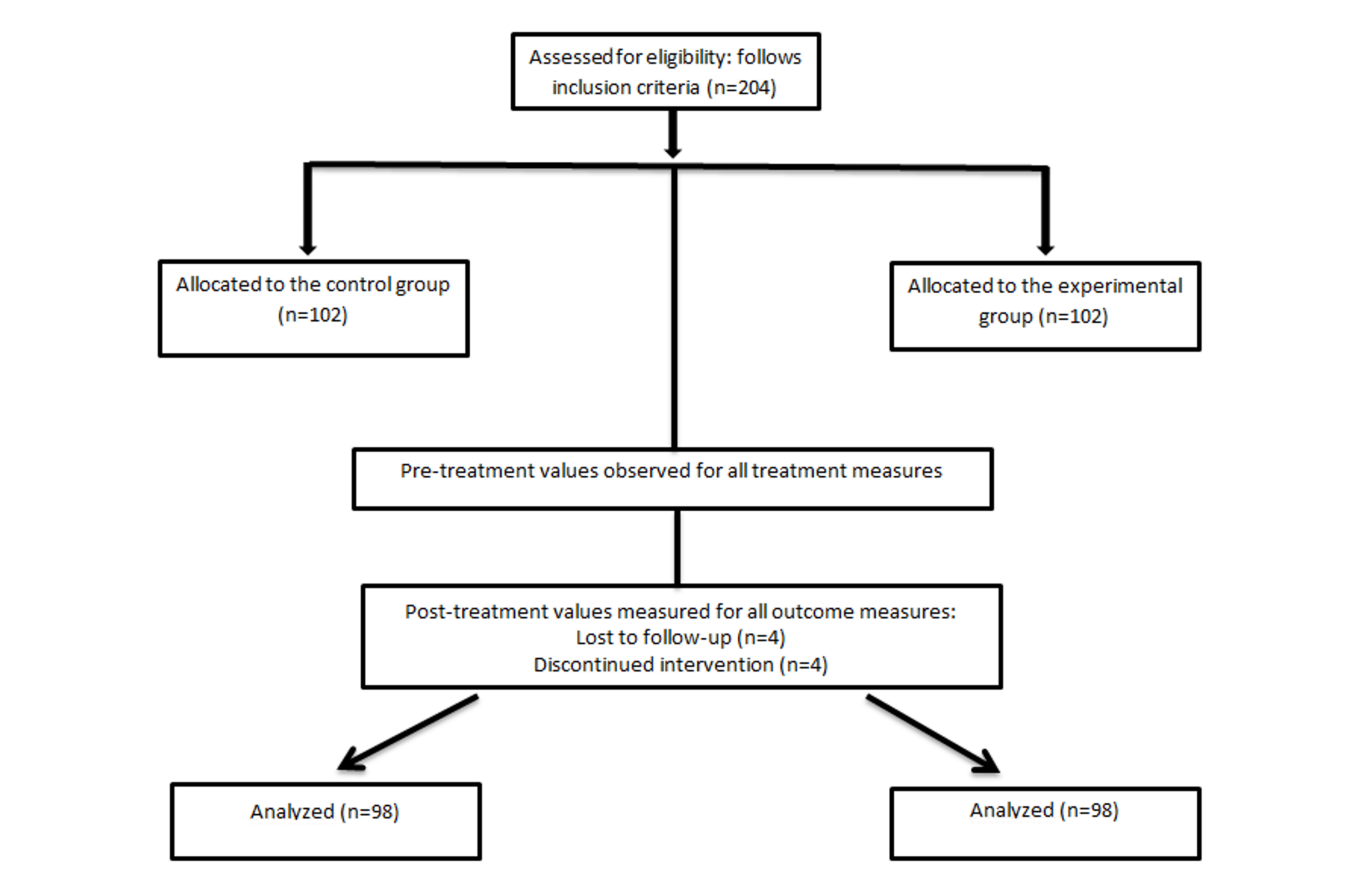
Study flowchart

A total of 204 participants were recruited and equally divided into two groups, with 102 individuals in each group. Subsequently, eight participants were lost to follow-up, resulting in 196 subjects who completed the study. Figure 1 explains the study flowchart. The intervention program spanned six weeks and was structured into five progressive phases, namely, warm-up, activation, stabilization, strengthening, and functional performance, concluding with cool-down exercises. Pre-intervention assessments included the Visual Analogue Scale (VAS), range of motion (ROM), manual muscle testing (MMT), and Knee Injury and Osteoarthritis Outcome Score (KOOS). Both land-based and aquatic exercises were incorporated into the protocol. Participants who met the inclusion and exclusion criteria were randomly allocated to Group A or Group B using a sealed envelope method. Group A received conventional physiotherapy, while Group B underwent CKCE using a customized knee brace. The customized knee brace used was a valgus unloader orthosis specifically designed to reduce medial compartment loading. It was fabricated using thermoplastic material based on each patient's anatomical measurements, with neoprene padding, Velcro straps for secure fixation, and metal hinges to allow controlled knee movement. The brace featured adjustable valgus angulation to provide optimal joint offloading. A certified orthotist performed the fitting to ensure correct alignment, comfort, and compliance. Both groups received baseline treatments, including hot moist packs and transcutaneous electrical nerve stimulation (TENS). The activation and stabilization phases were identical for both groups; however, the strengthening and functional task components were modified according to their respective group protocols [21,22]. For Group A, the warm-up phase included breathing exercises and basic ankle and toe mobility drills to improve circulation and prepare muscles for activity. The activation phase focused on early core and lower limb activation exercises such as pelvic bridging, tummy tucks, hamstring curls on a Swiss ball, and functional movements like marching, walking, and sit-to-stand, performed on both land and in water. Repetitions increased progressively. The stabilization phase introduced resistance-based exercises, including wall sits with ball presses and aquatic movements like step-ups and lateral walking to enhance neuromuscular control and joint stability. The strengthening phase emphasized progressive resistance training targeting major muscle groups (quadriceps, hamstrings, adductors) through isometrics, leg raises, and resistance-band exercises, with increased repetitions and hold durations. The functional performance phase aimed to mimic daily activities and improve dynamic balance and coordination using exercises like ball kicks, bridge with marching, and reach-outs in various positions. Cool-down concluded with static stretching of key lower limb muscle groups to promote flexibility and aid recovery. Aquatic exercises were conducted in a therapy pool maintained at a water temperature of 32-34°C and a depth of 3.5-4 ft to allow partial weight-bearing. Each session lasted approximately 30 minutes, including a five-minute warm-up and cool-down period. All the exercises were done under the supervision of the physiotherapist.

The exercise protocol for Group B has been mentioned in Table 1.

**Table 1:** Exercise protocol for Group B

Type of exercise	Exercises	Position and setting	Repetitions/holds/minutes×sets (weeks 1→6)
Warm-up exercises	Deep breathing exercises	Supine	2 minutes
	Ankle toe movements, ankle rotations, toe tapping, toe curls	Supine	10×3→30×3
Activation phase (1-2 weeks)	Pelvic bridging	Supine	5×3→10×3
	Tummy tucks	-	5 second hold×10→10×10
	Swiss ball hamstring curls	Supine	5×3→10×3
	Sit-to-stand	Land	5×3→10×3
	Seated heel raises	Sitting on land	10×3→30×3
Stabilization phase (2-3 weeks)	Wall sits with ball press	Land	5×3→10×3
	Standing terminal knee extension with resistance band	Land	5×3→10×3
	Step-ups on pool stairs	Water	10 steps×3→30×3
	Aquatic resistive knee extensions	Water	5×3→10×3
	Lateral walking (crab walk)	Water	10 steps×3→30×3
Strengthening phase (3-5 weeks) with knee brace	Lunges	Land	5×3→10×3
	Squats	Land	5×3→10×3
	Step-ups	Land	5×3→10×3
	Leg press	Land	5×3→10×3
	Stationary bike	Land	3 minute→10 minute
Functional performance (5-6 weeks) with knee brace	Single-leg stance	Land	5-10 seconds×3→15×3
	Step-ups	Land	5-10 seconds×3→30×3
	Toe walking	Land	1 minute→3 minutes
	Heel walking	Land	1 minute→3 minutes
	Single-leg stance by holding wand and then without wand	Land	5-10 seconds×3→15×3
Cool-down	Stretching (quadriceps, hamstrings, piriformis, calf muscles)	Land	5-10 seconds×3→15×3

**Figure 2:**
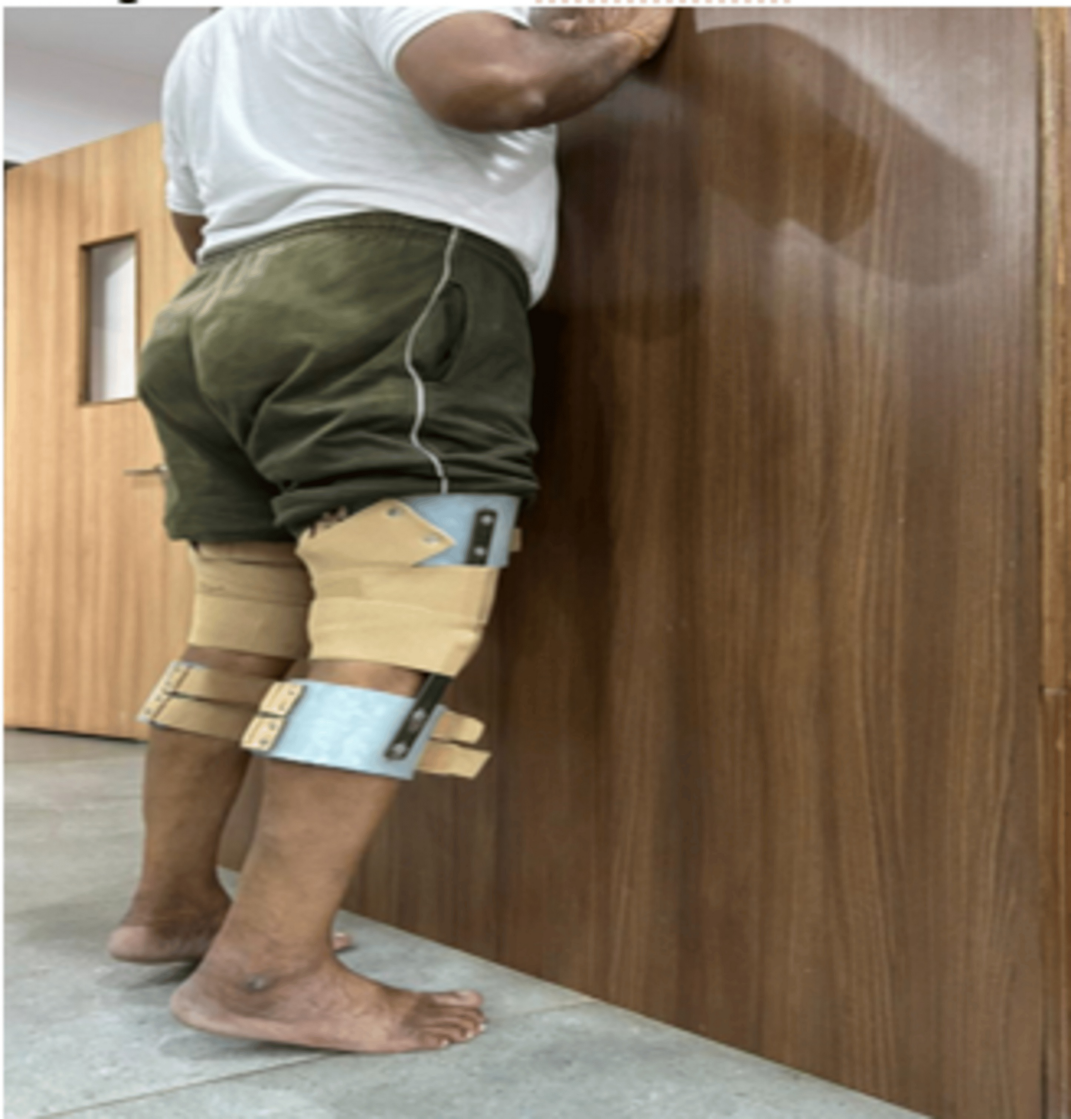
Heel raise with a customized knee brace

In Figure 2, the patient is seen doing heel raises with a customized knee brace.

**Figure 3:**
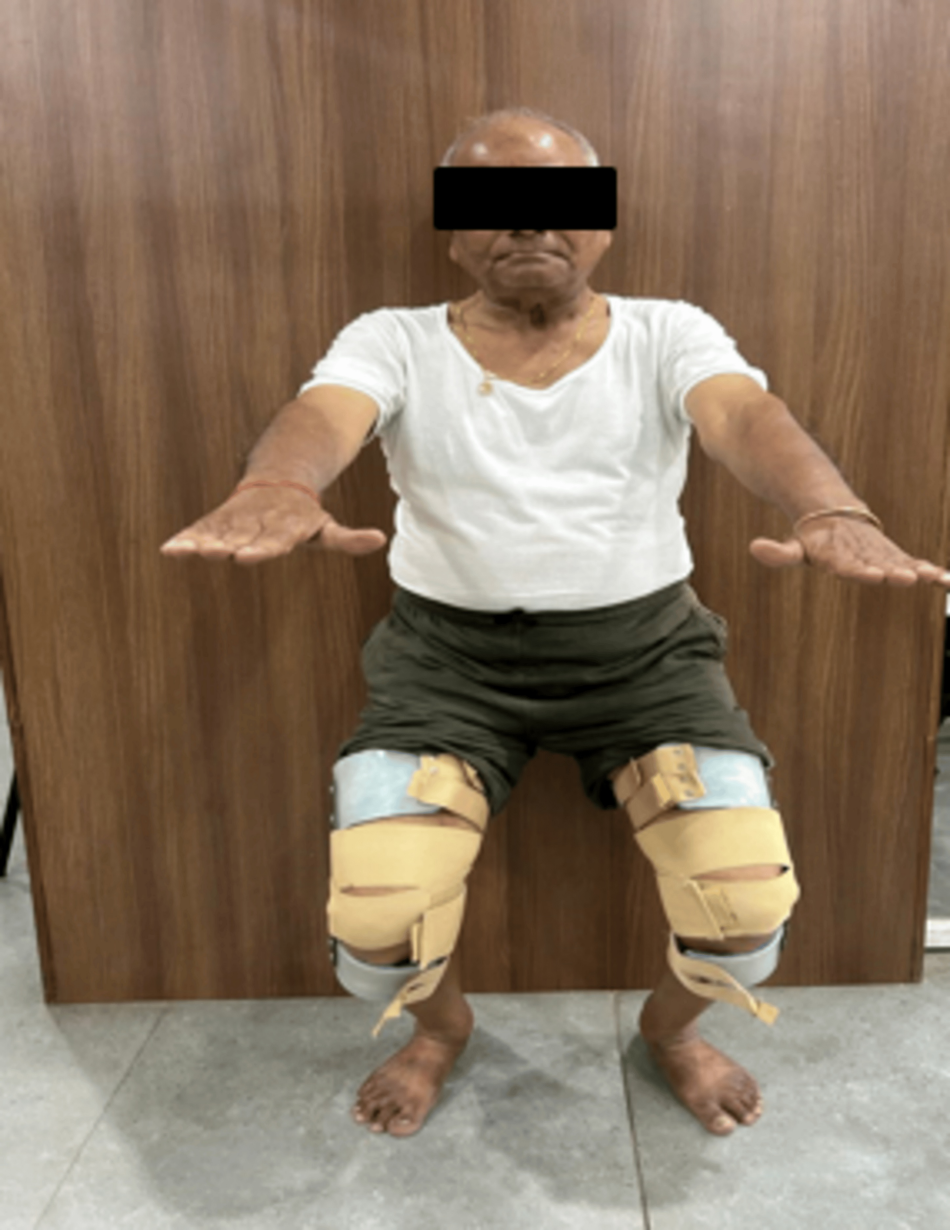
Squats with a customized brace

In Figure 3, the patient is seen doing squats with a customized knee brace.

Outcome measures

The outcome measures used in this study include the VAS, which quantifies pain intensity on a 10 cm line ranging from "no pain" to "worst pain", offering a reliable and simple method (95% CI=0.96-0.98) [23,24]. ROM assesses joint mobility using tools like goniometers, reflecting joint flexibility and treatment progress. ROM analysis is a reliable and valid method for measuring ROM, and it is a convenient and easy outcome measure for clinical trials and physiotherapy practice [25]. MMT evaluates muscle strength using the Oxford grading system (0-5 scale), where 0 indicates no movement and 5 represents full strength against maximum resistance [26]. The KOOS is a validated patient-reported questionnaire (ICC=0.91-0.99) covering five domains: pain, symptoms, activities of daily living, sports/recreation function, and knee-related quality of life [27,28]. 

Statistical analysis

Data analysis was performed both manually and using IBM SPSS Statistics for Windows, Version 26.0 (Released 2019; IBM Corp., Armonk, New York, United States). Descriptive statistics, including mean and standard deviation, were used to summarize the numerical data. Within-group comparisons of pre- and post-intervention values were analyzed using the paired t-test. Normality of continuous variables was assessed prior to further analysis. To evaluate changes across multiple time points, repeated-measures analysis was applied. A p-value threshold of <0.0001 was established to determine statistical significance, ensuring the reliability and rigor of the results. This approach allowed for comprehensive data interpretation by combining manual review with advanced statistical tools.

## Results

Table 2 shows the interpretation of demographic variables. The study involved 196 participants, evenly split into Group A and Group B (98 each), after excluding eight lost to follow-up. Most participants were aged 56-65, representing an older adult population. Females predominated in both groups, aligning with the higher incidence of knee OA in women. The majority had an obese BMI (30-34.9 kg/m²), with fewer falling in the normal range (25-29.9 kg/m²).

**Table 2:** Demographic variables BMI: body mass index

Variables	Group A	Group B
Age (years)		
45-55	36 (36.73%)	42 (42.86%)
56-65	62 (63.27%)	56 (57.14%)
Gender		
Male	31 (15.81%)	27 (13.77%)
Female	67 (24.18%)	71 (36.22%)
BMI		
25-29.9	25 (12.75%)	27 (13.77%)
30-34.9	73 (37.24%)	71 (36.22%)

Table 3 shows the within-group analysis of VAS. Pain was assessed using VAS at rest and during activity pre- and post-intervention in both Group A and Group B. Group A showed significant VAS reduction at rest (2.47±0.87 to 0.97±0.77) and during activity (6.84±1.31 to 3.42±1.07). Group B showed greater VAS reduction at rest (2.37±0.85 to 0.36±0.50) and during activity (7.29±1.20 to 1.23±1.06), with all values statistically significant (p<0.0001). Both groups improved, but Group B showed superior outcomes. Effect sizes were larger in Group B (d=2.82 (95% CI: 2.37-3.27) at rest and d=5.05 (95% CI: 4.42-5.68) during activity) compared to Group A (d=1.83 (95% CI: 1.49-2.17) at rest and d=2.91 (95% CI: 2.45-3.37) during activity). This indicates greater pain reduction in Group B, especially under functional load.

**Table 3:** VAS within-group analysis VAS: Visual Analogue Scale; SD: standard deviation; p<0.000: extremely significant

VAS	Pre-test (mean±SD)	Post-test (mean±SD)	Mean difference	P-value	T-value
At rest					
Group A	2.474±0.879	0.979±0.777	1.495	<0.0001	13.379
Group B	2.371±0.857	0.360±0.503	2.011	<0.0001	20.337
On activity					
Group A	6.846±1.319	3.428±1.075	3.418	<0.0001	25.133
Group B	7.295±1.203	1.234±1.063	6.061	<0.0001	39.169

Table 4 shows the within-group analysis of ROM. Both Group A and Group B showed statistically significant improvements (p<0.0001), with Group B demonstrating markedly greater gains across all measures. For knee flexion, Group A showed modest improvements: the right knee increased from 95.479±2.908 to 98.173±4.367 and the left knee from 99.632±6.270 to 102.775±7.892 (p=0.0010), with small to moderate effect sizes (right knee: d=0.694 and 95% CI: 0.288-1.100; left knee: d=0.432 and 95% CI: 0.058-0.805). In contrast, Group B showed substantial flexion gains: the right knee increased from 94.727±3.126 to 123.131±8.892 and the left knee from 101.295±6.855 to 127.948±5.000 (p<0.0001), with very large effect sizes (right knee: d=4.006 and 95% CI: 3.450-4.561; left knee: d=4.484 and 95% CI: 3.876-5.091). For extension, Group A improved from 2.653±1.567 to 1.959±1.592 on the right and from 2.448±1.520 to 1.513±0.912 on the left, with small to moderate effect sizes (right knee: d=0.438 and 95% CI: 0.150-0.726; left knee: d=0.700 and 95% CI: 0.408-0.991). Group B achieved greater reductions in extension: both knees improved to 0.479±0.721 (p<0.0001), with large effect sizes (right knee: d=1.746 and 95% CI: 1.374-2.118; left knee: d=1.648 and 95% CI: 1.278-2.018). These findings suggest that CKCE with a customized brace is significantly more effective in improving knee ROM than CKCE with a conventional brace. 

**Table 4:** ROM within-group analysis ROM: range of motion; SD: standard deviation; p<0.000: extremely significant

ROM	Pre-test (mean±SD)	Post-test (mean±SD)	Mean difference	P-value	T-value
Knee flexion					
Right knee					
Group A	95.479±2.908	98.173±4.367	2.694	0.0010	3.385
Group B	94.727±3.126	123.131±8.892	28.404	<0.0001	31.029
Left knee					
Group A	99.632±6.270	102.775±7.892	3.143	0.0010	3.388
Group B	101.295±6.855	127.948±5.000	26.653	<0.0001	28.596
Knee extension					
Right knee					
Group A	2.653±1.567	1.959±1.592	0.694	<0.0001	6.105
Group B	2.591±1.456	0.479±0.721	2.112	<0.0001	16.293
Left knee					
Group A	2.448±1.520	1.513±0.912	0.935	<0.0001	10.588
Group B	2.448±1.520	0.479±0.721	1.969	<0.0001	15.044

Table 5 shows the within-group analysis of MMT. Both groups showed significant strength improvements, with Group B exhibiting markedly greater gains. For knee flexors, Group A showed modest increases: the right knee improved from 3.653±0.478 to 3.897±0.650 (p=0.0011) and the left knee from 3.540±0.595 to 3.765±0.743 (p=0.0012), with small effect sizes (right knee: d=0.408 and 95% CI: 0.131-0.686; left knee: d=0.335 and 95% CI: 0.055-0.614). In contrast, Group B demonstrated substantial flexor strength improvements: the right knee from 3.520±0.691 to 4.734±0.584 and the left knee from 3.540±0.595 to 4.357±0.911 (p<0.0001), with large effect sizes (right knee: d=1.923 and 95% CI: 1.539-2.307; left knee: d=1.021 and 95% CI: 0.667-1.375). For knee extensors, Group A improved from 3.438±0.498 to 3.571±0.625 on the right and from 3.408±0.5341 to 3.540±0.577 on the left (p<0.0014), with small effect sizes (right knee: d=0.228 and 95% CI: 0.019-0.438; left knee: d=0.236 and 95% CI: 0.027-0.445). Group B exhibited marked improvements: the right knee from 3.408±0.543 to 4.551±0.499 and the left knee from 3.397±0.4920 to 4.571±0.497 (p<0.0001), with very large effect sizes (right knee: d=2.133 and 95% CI: 1.738-2.528; left knee: d=2.336 and 95% CI: 1.929-2.744). These findings indicate the superior impact of the intervention in Group B on improving knee muscle strength.

**Table 5:** MMT within-group analysis MMT: manual muscle testing; SD: standard deviation; p<0.000: extremely significant

MMT	Pre-test (mean±SD)	Post-test (mean±SD)		P-value	T-value
Knee flexors					
Right knee					
Group A	3.653±0.478	3.897±0.6500	0.244	0.0011	3.3703
Group B	3.520±0.691	4.734±0.584	1.214	<0.0001	12.879
Left knee					
Group A	3.540±0.595	3.765±0.743	0.225	0.0012	3.335
Group B	3.54±0.595	4.357±0.911	0.817	<0.0001	9.746
Knee extensors					
Right knee					
Group A	3.438±0.498	3.571±0.625	0.133	0.0013	3.309
Group B	3.408±0.543	4.551±0.499	1.143	<0.0001	15.452
Left knee					
Group A	3.408±0.5341	3.540±0.577	0.132	0.0014	3.292
Group B	3.397±0.4920	4.571±0.497	1.174	<0.0001	16.505

Table 6 shows the within-group analysis for KOOS. Significant improvements were observed in all five domains, namely, pain, symptoms, ADL, sports, and quality of life, with Group B demonstrating consistently greater gains. For pain, Group A improved from 52.561±5.189 to 66.543±5.200 (p<0.0001), with Cohen's d=2.706 (95% CI: 2.203-3.209), while Group B improved from 52.807±6.238 to 73.210±4.659 (p<0.0001), with a larger effect size (d=3.717 (95% CI: 3.146-4.288)). In symptoms, Group A increased from 51.350±4.842 to 65.350±6.317 (p<0.0001) (d=2.468 (95% CI: 1.972-2.963)), whereas Group B improved from 51.543±4.602 to 69.017±7.429 (p<0.0001) (d=2.842 (95% CI: 2.328-3.356)). In ADL, Group A improved modestly from 52.350±5.156 to 56.000±9.140 (p=0.0015) (d=0.507 (95% CI: 0.194-0.820)), while Group B showed a substantial increase from 53.017±4.910 to 73.894±6.750 (p<0.0001), with d=3.702 (95% CI: 3.132-4.272). In the sports domain, Group A improved slightly from 15.315±20.217 to 17.894±21.957 (p = 0.0027) (d=0.127 (95% CI: 0.040-0.214)), while Group B showed dramatic gains from 15.315±20.217 to 44.622±9.429 (p<0.0001) (d=1.746 (95% CI: 1.373-2.118)). For quality of life, Group A improved from 42.842±6.956 to 48.192±11.689 (p=0.0001) (d=0.547 (95% CI: 0.233-0.861)), while Group B achieved a remarkable increase from 48.228±6.801 to 83.421±4.982 (p<0.0001), with an exceptionally large effect size (d=5.982 (95% CI: 5.317-6.646)). These results highlight the superior efficacy of the intervention in Group B across all KOOS domains.

**Table 6:** KOOS within-group analysis KOOS: Knee Injury and Osteoarthritis Outcome Score; SD: standard deviation; p<0.000: extremely significant

KOOS	Pre-test (mean±SD)	Post-test (mean±SD)	Mean difference	P-value	T-value
Pain					
Group A	52.561±5.189	66.543±5.20	13.982	<0.0001	15.976
Group B	52.807±6.238	73.210±4.659	20.403	<0.0001	18.760
Symptoms					
Group A	51.350±4.842	65.350±6.317	14.000	<0.0001	14.252
Group B	51.543±4.602	69.017±7.429	17.474	<0.0001	16.832
Activities of daily living					
Group A	52.350±5.156	56.0±9140	3.650	0.0015	3.350
Group B	53.017±4.91	73.894±6.750	20.877	<0.0001	20.232
Recreation and sports					
Group A	15.315±20.217	17.894±21.957	2.579	0.0027	3.142
Group B	15.315±20.217	44.622±9.429	29.307	<0.0001	10.793
Quality of life					
Group A	42.842±6.956	48.192±11.689	5.350	0.0001	4.148
Group B	48.228±6.801	83.421±4.982	35.193	<0.0001	43.427

Table 7 shows the between-group analysis for all outcomes. Post-test results showed that Group B achieved significantly greater improvements than Group A across all outcome measures (p<0.0001). Group B experienced reduced pain (VAS), enhanced knee ROM, and increased muscle strength in both flexors and extensors. KOOS scores further indicated superior pain relief, symptom control, functional ability, sports participation, and quality of life. These findings highlight the greater effectiveness of the intervention used in Group B for managing bilateral medial compartment knee OA.

**Table 7:** Between-group analysis for all components VAS: Visual Analogue Scale; ROM: range of motion; MMT: manual muscle testing; KOOS: Knee Injury and Osteoarthritis Outcome Score

	Post-test (mean±SD) Group A	Post-test (mean±SD) Group B	Mean difference	P-value	T-value
VAS					
On rest	0.979±0.777	0.360±0.503	-0.619	<0.0001	6.6203
On activity	3.428±1.075	1.234±1.063	-2.194	<0.0001	14.366
ROM					
Knee flexion					
Right knee	98.173±4.367	123.131±8.892	24.958	<0.0001	24.940
Left knee	102.775±7.892	127.948±5.000	25.173	<0.0001	23.673
Knee extension					
Right knee	1.959±1.592	0.479±0.721	-1.480	<0.0001	8.383
Left knee	1.513±0.912	0.479±0.721	-1.034	<0.0001	8.804
MMT					
Knee flexors					
Right knee	3.897±0.6500	4.734±0.584	0.837	<0.0001	9.482
Left knee	3.765±0.743	4.357±0.911	0.592	<0.0001	4.9852
Knee extensors					
Right knee	3.571±0.625	4.551±0.499	0.980	<0.0001	12.130
Left knee	3.540±0.577	4.571±0.497	1.031	<0.0001	13.402
KOOS					
Pain	66.543±5.20	73.210±4.659	6.667	<0.0001	9.435
Symptoms	65.350±6.317	69.017±7.429	3.667	<0.0001	3.7226
Activities of daily living	69.017±7.429	73.894±6.750	4.877	<0.0001	4.809
Sports and recreation	17.894±21.957	44.622±9.429	26.728	<0.0001	11.072
Quality of life	44.622±9.429	83.421±4.982	38.799	<0.0001	36.016

## Discussion

The findings of the present study demonstrate that a structured CKCE program, when combined with the use of a customized knee brace, leads to significant improvements in both pain levels and functional capacity among individuals with bilateral medial compartment knee OA. These outcomes align with previous research supporting the efficacy of CKCE in managing knee OA. CKCE facilitates co-contraction of periarticular muscles, enhancing joint stability and promoting more balanced loading across the knee. The addition of a customized valgus unloader brace likely amplified these effects by externally correcting malalignment and reducing medial compartment stress, thereby improving joint mechanics during exercise. Given the degenerative and compensatory nature of bilateral knee OA, simultaneous strengthening with mechanical support may have helped correct movement asymmetries and enhance overall functional performance. The synergy between targeted exercise and external bracing appears to play a crucial role in optimizing outcomes in this population.

Dewi et al. examined the effectiveness of CKCE in patients with knee OA and reported significant improvements in pain reduction and functional abilities following the exercise intervention. Similarly, in the present study, both Group A and Group B showed highly significant reductions in pain, with VAS scores at rest and during activity improving markedly (p<0.0001 for both groups). These results support Dewi et al.'s findings that CKCE promotes pain relief and functional improvement. However, while Dewi et al.'s study primarily focused on pain during walking tasks, our research additionally demonstrated improvements in pain at rest, suggesting broader benefits of CKCE training in individuals with bilateral medial compartment knee OA [29].

Lin et al. reported that CKCE improved quadriceps strength and self-reported functional performance in individuals with knee OA. In alignment with these findings, our study also observed meaningful improvements in muscle strength following the intervention. Participants in both groups exhibited strength gains, with the group receiving CKCE combined with a customized knee brace showing more pronounced progress. Additionally, improvements were evident in multiple domains of the KOOS, including pain, symptoms, and ADL. Notably, our study extended these observations by demonstrating further enhancements in sports and recreation participation, as well as knee-related quality of life, highlighting the broader functional benefits of the combined intervention strategy [30].

Adegoke et al. compared open and CKCE and reported that CKCE led to superior improvements in ROM and pain reduction in individuals with knee OA. Our findings are consistent with their conclusions, as participants in our study also experienced meaningful improvements in knee flexion and a notable reduction in pain intensity following the intervention. While Adegoke et al.'s study focused on individuals with unilateral OA, our research contributes further evidence by demonstrating the effectiveness of CKCE in patients with bilateral medial compartment involvement. This highlights the broader applicability of CKCE in managing more extensive degenerative changes in the knee [31].

Jain and Shinde conducted a randomized controlled trial comparing the effects of open kinetic chain exercises and CKCE on dynamic balance and ROM in individuals with knee OA. While both exercise approaches led to improvements in ROM and balance within their respective groups, the differences between groups were not found to be statistically significant. In contrast, our study demonstrated marked improvements in both knee flexion and extension ROM across groups, with the CKCE and customized brace group showing more substantial gains. Furthermore, enhancements in pain reduction and functional performance were clearly reflected through both subjective and objective outcome measures. These findings support the notion that although both OKCE and CKCE are effective, CKCE may provide more comprehensive benefits in improving mobility and functional capacity in patients with knee OA [32].

In a randomized controlled trial, Fadhilah and Widodo compared the effects of open kinetic chain exercises and CKCE on the quadriceps-to-hamstring (Q/H) ratio, muscle thickness, and functional ability in individuals with knee OA. The study reported that CKCE led to greater improvements in the Q/H ratio and quadriceps muscle development, while open kinetic chain exercise was more beneficial in enhancing hamstring strength. In our study, both groups exhibited notable improvements in muscle strength, with the group performing CKCE with a customized brace demonstrating more pronounced gains in both knee flexor and extensor strength. These results are consistent with Fadhilah and Widodo's findings and further support the use of CKCE as an effective strategy for improving muscle balance, strength, and overall functional outcomes in patients with knee OA [33].

Strengths

This study possesses several notable strengths. The relatively large sample size (n=196) enhances the statistical power and generalizability of the findings, especially significant given that many physiotherapy trials are limited by small cohort sizes. Furthermore, the combined intervention, structured CKCE along with a customized valgus unloading knee brace, represents a novel and clinically relevant approach for managing bilateral medial compartment knee OA. The detailed and replicable protocol, including both land-based and aquatic components, improves the feasibility of implementation across diverse clinical settings. The observed improvements were statistically significant across all primary outcomes (VAS, ROM, MMT, KOOS), with high t-values and p-values consistently below 0.0001, reinforcing the robustness of the findings.

Limitations

Despite its strengths, this study has certain limitations. It was conducted at a single institution, which may limit the generalizability of the findings to broader populations. The intervention period was relatively short, spanning only six weeks, and no long-term follow-up was conducted.

## Conclusions

This study highlights the clinical value of incorporating CKCE along with a customized knee brace in the rehabilitation of individuals with bilateral medial compartment knee OA. The combined intervention significantly reduces pain, enhances joint mobility, increases muscle strength, and improves functional performance more effectively than conventional physiotherapy alone. The customized knee brace provides external support by correcting malalignment and offloading the medial compartment, while CKCE enhances joint stability, neuromuscular control, and proprioception. Together, they promote better mobility, functional independence, and overall quality of life. Given these measurable benefits, CKCE combined with bracing should be considered a vital component of conservative management strategies for knee OA.
